# Supplementary material for: Active site specificity profiling datasets of matrix metalloproteinases (MMPs) 1, 2, 3, 7, 8, 9, 12, 13 and 14
Source: Data Brief. 2016 Feb 22;7:299–310. doi: 10.1016/j.dib.2016.02.036 (PMC4777984; doi:10.1016/j.dib.2016.02.036)
Supplement: Supplementary file 10 — Supplementary material [file mmc10.zip › WebPICS_hMMP9_G_1%/P2.html]

 

PICS results


|  |  |
| --- | --- |
| **P2\_A**  19 in 146 sites   13.0 %    effects > 10 perc. pnts.  (vice-versa in brackets)  P3\_V: 17.4 (25.5)   P1prime\_I: 16.5 (14.3)   P2prime\_A: 12.2 (17.8) |  |
| **P2\_C**  2 in 146 sites   1.4 %    effects > 10 perc. pnts.  (vice-versa in brackets)  P2prime\_C: 97.3 (48.6) |  |
| **P2\_K**  20 in 146 sites   13.7 %    effects > 10 perc. pnts.  (vice-versa in brackets)  P1\_A: 13.6 (11.3)   P1\_R: 14.7 (19.6)   P2prime\_K: 12.7 (14.1)   P2prime\_V: 16.3 (16.3)   P3prime\_G: 18.8 (12.1) |  |
| **P2\_Q**  6 in 146 sites   4.1 %    effects > 10 perc. pnts.  (vice-versa in brackets)  P1\_Q: 12.6 (12.6)   P1prime\_Q: 25.1 (12.6)   P2prime\_R: 38.4 (13.5) |  |
| **P2\_R**  20 in 146 sites   13.7 %    effects > 10 perc. pnts.  (vice-versa in brackets)  P1prime\_V: 11.8 (19.6) |  |
| **P2\_Y**  5 in 146 sites   3.4 %    effects > 10 perc. pnts.  (vice-versa in brackets)  P1\_H: 16.6 (16.6)   P2prime\_Q: 15.9 (13.3) |  |
